# Factors associated with glycaemic control in Singapore children and young people with diabetes

**DOI:** 10.1186/1687-9856-2013-S1-P18

**Published:** 2013-10-03

**Authors:** Ngee Lek, Angela Hui, Bixia Ang, Christine Chua, Suzanne Goh, Pei Kwee Lim, Joyce Lim, Rashida Vasanwala, Fabian Yap

**Affiliations:** 1 KK Women’s and Children’s Hospital, Singapore

## 

Data from follow-up studies of DCCT (EDIC) [1,2] and UKPDS [3] suggest that tight glycaemic control in the early years after diagnosis of diabetes may delay the onset and development of diabetes-related complications. This phenomenon of “metabolic memory” [4] is particularly relevant for patients who are diagnosed during childhood and adolescence because of potentially longer lifetime duration of diabetes. We conducted the present study to identify the factors associated with glycaemic control in Singapore children and young people with diabetes.

A cross-sectional analysis of data from our Paediatric Diabetes Service database as at the end of 2011 was performed. We included 125 patients (57 male; 46%), age <20 years and diagnosed with Type 1 (T1D) or Type 2 (T2D) diabetes for >1 year duration, who had attended clinic at least twice during 2011, and whose mean HbA1c measured at their clinic attendances was either good (HbA1c <8%; Group 1, n=69) or poor (HbA1c >10%; Group 2, n=56). The following factors in the two groups were compared: patient characteristics (gender, ethnicity, current age, age at diagnosis, duration of diabetes, type of diabetes, family history); medical treatment prescribed (insulin, oral agents, both); and extent of resource utilization (diabetes clinics, medical social worker (MSW) input, dietician reviews). Patients whose glycaemic control was intermediate (8% ≤ HbA1c ≤ 10%), and those with other types of diabetes (non-T1D and non-T2D), medical co-morbidities, or who had transitioned to the Adult Diabetes Service during 2011, were excluded.

Compared to Group 1 (good control) patients, Group 2 (poor control) patients were more likely to be non-Chinese (52% vs 24%; p=0.01) and to have received MSW input (30% vs 9%; p=0.002) and attended more diabetes clinics in 2011 (4.4±1.3 vs 3.5±1.0 times; p<0.001) – see Table 1. Multivariate analyses found that these three factors remained independently predictive of poor glycaemic control. There was no statistically significant difference between the two groups in all the other factors examined.

Ethnicity and utilization of healthcare and social services were factors associated with glycaemic control in Singapore children and young people with diabetes. These findings suggest that there are paediatric diabetes patient groups in which the desired outcome was not achieved despite providing higher levels of currently available support. Therefore, we need to devise new ways of improving the management of these at-risk patients.
